# Indents

**Published:** 1920-01

**Authors:** 


### INDENTS

**☞Brief Articles of Technical or Scientific Value will receive notice and comment. Contributions invited.**

---

A Quick Filter. A nice and quick way to filter water for hypodermic use is to boil the water in a teaspoon over a common flame, place a small piece of absorbent cotton in the spoon, and draw the liquid into the syringe through the cotton. By so doing you get a perfectly clear solution.—*Dental Surgeon.*

---

Plate Polishing. Metal plates will take on a very high luster when a little aqua ammonia is used in mixing the chalk instead of water. Vulcanite will take on a mirror polish if the finishing is done by the use of tin oxide made into a paste with glycerine. In either case a clean buff should be chosen, and kept exclusively for this purpose.—*Dental Record.*

---

Roach Incisor Replacement. To replace two upper incisors by a removable bridge: Cast two lingual plates for the cuspids and attach for anchorage two finger-wire clasps to the distal angles of the plates so that the wire clasps will pass over the incisal surfaces in the interproximal spaces and terminate in the labial embrasures. The two cribs are then united by a wire extending from one cuspid to the other lying on the lingual surfaces of the incisors. To this wire the laterals are attached, making a removable bridge without mutilation.

---

Dental Education. Not that we all entirely agree with the education of today. Some of us look with a little concern on the tendency which certainly exists in some quarters to broaden the curriculum at the expense of that practical work which constitutes after all the most important part of dentistry, the training for which seems to us in danger of being made perfunctorily short. It would be a sorry thing if a widened scope of learning should endanger concentration on that fine manipulative work which is demanded at the hands of anyone who is to be a dentist worthy of the name. It is not always the brilliant student who goes on to do brilliant work as a practitioner. For that one needs not only book knowledge, not only a broad foundation and its ancillary sciences, not only character and conscience, not only gentleness and cheerfulness ("these," says Robert Louis Stevenson, "come before all morality; they are the perfect duties," and if that is true of anybody, it must be true of the dentist); one needs above all the ability to perform delicate and often very difficult operations with delicacy and precision, only to be acquired by persistent and painstaking practice. And that, when all is said and done, is what makes, or should make, lifelong students of us all. It is a truism, but one which bears restating from time to time, that only those can achieve real success who keep an open and inquiring mind, who are always ready to learn, and whose high endeavor to the last is at all costs to do good work for its own sake.—*British Dental Journal.*

---

CLEAN FOOD. Philanthropic societies of New York have been providing pure food, cooked in clean utensils, for the poor children, so they might have food to sustain them in their work. These little bodies have to grow, and their mentality has to grow—every part of them has to grow. The water and the food have to go into the mouth, and have to be mixed there, and therefore the condition of the mouth will have much to do with the condition of the food before it is taken into the stomach. Dr. Goadby of England, speaking before a body of surgeons and telling them what they must do to have perfect success after major surgical operations, spoke to them as follows: "You must examine the mouths of your patients. You would not allow your patients to apply their lips to a running ulcer on the arm, and swallow the pus, yet you know that broken-down bone tissue is more poisonous and irritating to the digestive organs than the matter that comes from a running ulcer. Then how can you allow them to retain a broken-down tooth in the mouth, when they are swallowing this matter all the time?''

If this is true in regard to patients who have passed through a major surgical operation, how much more true must it be when applied to young, growing children. The work of conserving the health of the children is not finished by government and municipal inspection of foods and water, but must be carried on to the mouth of the child, and provision must be made to provide the necessary means to accomplish this.—*Dental Cosmos* for November.

---

Poison Labels. Hereafter the skull and bones symbol, and the word ''Poison'' must be printed in red ink on all labels affixed to wholesale and retail packages of completely denatured alcohol, according to instructions received today by Collector of Internal Revenue Justus S. Wardell from the Department. This action, the report states, has been taken in view of the grave and extended abuses of the use of completely denatured alcohol.

In his instructions the commission says:

''Reports recently received in the Bureau establish that completely denatured alcohol is being used extensively for bathing and rubbing purposes. This is contrary to the law and regulations and such uses can not be tolerated, as the completely denatured alcohol is highly injurious to the skin and animal tissue.

''It is also established that completely denatured alcohol is being sold by irresponsible dealers under such circumstances as to assure them that it is being used for beverage purposes. Where it is so used for any length of time blindness inevitably ensues, and the continued use can only result in death."

Collector Wardell stated that wherever such use of the denatured alcohol as above indicated is discovered within his jurisdiction ultimate penalties provided by law will be invoked.

---

THE NESBITT REMOVABLE BRIDGE: MODIFIED TECHNIC.

Take an impression of the abutment teeth after they have been ground to clear the occlusion. Also take a wax bite at this time; varnish as above, and run the model in Standard investment compound and allow this to harden well. Now take an impression of the abutment teeth separately or individually, with one of the small hinged trays that Dr. Roach uses for this purpose. (A small door hinge can be bent into shape for this.) This has the advantage of allowing the tray to be opened after the impression has set in the mouth, thus facilitating the removal of the same from the tooth without too much mutilation. Varnish, and run these impressions up in the investment that is to be used in the casting of the clasp. After they have hardened, remove and wax up the clasp on these forms. Soak the same in water, and attach sprue wires to the wax forms, which have been left on the tooth form of inlay investment compound; then put all on the sprue-former, place ring around them, invest in the usual manner and cast. After clasps are cast, clean them and remove all nodules and rough places, and place them on the other model that has been run in Standard investment compound at the beginning. Assuming that the dummies are ready to place on the model for soldering, wax the same to the dummies. Now soak this Standard model in water, and it will be very easily trimmed down small enough to admit of being placed in a soldering ring and invested, for final attachment of the clasp to the dummies, when the bridge will be complete. This method does away with the teasing off of the wax forms from the model, and also does away with having to remove the clasps from the model after placing them there the first time, thus doing away with the liability of making some mistake by making too many changes.—W. M. Curt, *Texas Dental Journal.*

---

ORAL HYGIENE AS AN ECONOMIC FACTOR. In Greater New York we were requested to make an examination of 2,104 girls in one of the high schools. The findings were recorded on specially prepared charts which saved much time. The physical director of the school and her assistants made a study of these records and made a report to us of considerable interest. They found that the best room average was 1.3 cavities per girl. The worst room average was 3.5 cavities per girl. (Every girl in this room was a "held back," or one who had failed to pass her last examination.) Do not go away with the idea that I believe that because these girls had some cavities in their teeth they could not pass their examination. Not at all; but those who are careless with the hygienic condition or cleansing of the mouth are more than apt to be careless with their appearance, their rooms, and their studies; in other words, they are generally careless in all they do. Those who are careful of their personality and their appearance are likely to be careful of their studies and their dress and everything else.

Dr. A. C. Fones, of Bridgeport, Conn., had much difficulty in starting dental work in the schools, but finally succeeded in securing an appropriation, a small one, but something to start with. Dr. Fones and some of his dental friends established dental clinics for prophylactic purposes, teaching the children how to properly use the toothbrush and also giving illustrated talks.

In previous years, forty per cent. of the amount of the budget for educational purposes in Bridgeport was used for the re-education of children. After five years of dental work and teaching the children how to care for their mouths, only seventeen per cent. of the budget was used for the re-education of the children. As the cost for educating one child for one year in New York city is something like $30, you can realize what a tremendous saving it will be to the taxpayers when the authorities awake to an appreciation of the value of mouth hygiene.—Dr. Hyatt, in *Cosmos,* for November.

---

REMOVAL OF RUST FROM STEEL INSTRUMENTS. To remove rust from steel instruments, Sangu recommends placing them over night in a saturated solution of zinc chlorid. The rust disappears through reduction. On removing the instruments they are to be rinsed in clear water, placed in hot soda and soap solution, and dried. It is also advantageous to polish with absolute alcohol and chalk.—*Pacific Medical Journal,* per *Dental Surgeon.*

---

RELATIONSHIP OF DENTAL ABSCESSES AND TOXEMIAS OF PREGNANCY. Eight cases are reported by Loomis in which he is convinced that all the evidence is at hand to connect abscesses of the teeth with manifestations of toxemias of pregnancy. He suggests that this relationship be borne in mind and that extraction of abscessed teeth is indicated.—*California State Journal of Medicine,* November.

---

EXCLUSION OF MOISTURE IN ITS RELATION TO ASEPSIS. The first rule of modern surgery is asepsis. As applied to pulp treatment, this means that the field of operation should be treated as though it were a surgical wound. The immediate neighborhood should be maintained in an aseptic condition during each operation.

Nothing carrying infection should be permitted to enter this field. In no case should saliva be allowed to enter the pulp chamber from the beginning of the first treatment until after the root canals are filled. This may be done by so simple a technique that there is no reason why it should not be carried out to the finest detail except in a very limited number of cases which present unusual difficulties.

Under this plan, asepsis in pulp treatment requires:

1. The mechanical procedure to secure cleanliness before applying the rubber dam. This may be done by first spraying the mouth with an antiseptic solution; then by cleansing and disinfecting the crevices and gingiva of the teeth to which the rubber dam is to be applied.

N. B.—If this precaution is omitted infectious material is forced by the rubber and ligatures under the gum margin, where it may remain for hours. The interruption of the circulation of the blood favors infection of the weakened tissue.

2. The disinfection of the field by hydrogen dioxide, followed by thymol alcohol.

3. Application of the rubber in such a way as to exclude all moisture from the field of operation.

4. Sterilization of the field of operation as best possible by swabbing the surfaces of the teeth with a good disinfectant, such as iodine.—*Dominion Dental Journal.*

---

CEMENTING A CROWN OR BRIDGE. To mix cement stiff enough to get prompt setting, drill a small vent-hole in the occlusal surface of the crown for escape of excess cement. This enables easy and accurate placement and avoids the possibility of grinding the crown to get correct occlusion afterwards. The vent-hole is easily corrected by drilling out the cement and filling with gold and much time and annoyance saved.—F. W. Sage, in *Summary.*

---

THE PHYSICIAN AND HIS DUTIES TO SOCIETY. This address by Aráoz Alfaro at the commencement exercises of the medical school has been widely copied. In conclusion he remarked that if any of the recent graduates did not feel inspired by love for humanity and sympathy for the pain of others, and did not feel capable of self-abnegation and sacrifice, he urged them to withdraw from the ranks of the profession and seek other spheres of activity more propitious for an easy, prosperous life. "The joys of the practice of medicine are moral ones. It is superior to other careers by the high spirit of sacrifice which inspires it. Without altruism, without love for mankind, it will be a dreary burden to carry. Any other profession or livelihood would be preferable to it in this case." He gave a few aphorisms, the last one warning to distrust already published opinions. "Try to correct them or verify them. * * *" He advised the graduates to go into politics —"Not petty local politics, or subservience to men in office, but the politics of educational progress, of economic and social reforms, movements for hygiene and public health, the politics of institutional truth and wise prevision, which will carry the country far on the road of progress."—*Journal A. M. A.*, December 13, 1919.

